# Effects of whole-body cryotherapy on 25-hydroxyvitamin D, irisin, myostatin, and interleukin-6 levels in healthy young men of different fitness levels

**DOI:** 10.1038/s41598-020-63002-x

**Published:** 2020-04-10

**Authors:** Ewa Śliwicka, Tomasz Cisoń, Anna Straburzyńska-Lupa, Łucja Pilaczyńska-Szcześniak

**Affiliations:** 1 Poznan University of Physical Education, Department of Physiology and Biochemistry, Poznań, Poland; 2 State University of Applied Science in Nowy Sącz, Department of Physiotherapy, Nowy Sącz, Poland; 3 Poznan University of Physical Education, Department of Physical Therapy and Sports Recovery, Poznań, Poland; 4 State University of Applied Sciences in Kalisz, Faculty of Rehabilitation and Sport, Kalisz, Poland

**Keywords:** Biochemistry, Physiology

## Abstract

Skeletal muscle and adipose tissue play an important role in maintaining metabolic homeostasis and thermogenesis. We aimed to investigate the effects of single and repeated exposure to whole-body cryotherapy in volunteers with different physical fitness levels on 25-hydroxyvitamin D (25(OH)D) and myokines. The study included 22 healthy male volunteers (mean age: 21 ± 1.17 years), who underwent 10 consecutive sessions in a cryogenic chamber once daily (3 minutes, −110 °C). Blood samples were collected before and 30 minutes and 24 hours after the first and last cryotherapy sessions. Prior to treatment, body composition and physical fitness levels were measured. After 10 cryotherapy treatments, significant changes were found in myostatin concentrations in the low physical fitness level (LPhL) group. The 25(OH)D levels were increased in the high physical fitness level (HPhL) group and decreased in the LPhL group. The HPhL group had significant changes in the level of high-sensitivity interleukin-6 after the first treatment. The LPhL group had significant changes in 25(OH)D, irisin, and myostatin levels after the tenth treatment. Our data demonstrated that in healthy young men, cryotherapy affects 25(OH)D levels, but they were small and transient. The body’s response to a series of 10 cryotherapy treatments is modified by physical fitness level.

## Introduction

Whole-body cryotherapy (WBC) (also referred to as whole-body cryostimulation) has been used for many years in Europe. Initially, due to its analgesic and anti-inflammatory effects, it was empirically applied as a symptomatic adjunct therapy in rheumatic diseases (rheumatoid arthritis, ankylosing spondylitis, and fibromyalgia)^1–4^. In recent years, there has been a growing interest in the possibility of administering systemic cryotherapy to athletes and physically active individuals to improve recovery of injured muscles following exercise^5–7^ and enhance athletic performance^8^.

The effects of acute or chronic exposure to low temperatures on the human body and the resulting physiological reactions are continuously being researched. Low temperature exposure affects several biological reactions in the body, which are mediated by the activation of the hypothalamic-pituitary-adrenal axis and the sympathetic nervous system, along with an increased secretion of cortisol and catecholamines^9^. Bleakley *et al*.^10^ concluded that WBC could have a potentially beneficial effect on inflammatory mediators, antioxidant capacity, and autonomic function during recovery. Cryotherapy has also been shown to play a preventative role against the harmful effects of inflammation and pain caused by exercise^6^. Lombardi *et al*.^6^ noted that WBC does not always lead to beneficial biochemical changes; however, it may improve the final clinical status of the individual by reducing the pain experienced during post-exercise recovery.

Most of the research conducted on WBC focuses on the acute effects of single or repeated exposure to WBC treatment in athletes. There is little data on the length of time that the physiological effects of WBC are maintained. In our previous study, we showed that changes in selected blood biochemical parameters in hockey players returned to the baseline value or a value slightly above this one week after the end of therapy^11^. Moreover, some authors emphasise that the efficacy and safety of WBC have not been demonstrated^2,12^.

During cold exposure, a mechanism to minimise heat loss (vasoconstriction of vessels in the skin) and an increase in heat production are activated^13^. Brown adipose tissue (BAT) and skeletal muscle are involved in shivering- and non-shivering- thermogenesis^14^. Cold exposure results in the activation of brown fat via the sympathetic nerves, resulting in regulatory heat production by burning the energy substrates as heat rather than generating energy and storing it as fat^15^.

The physiological response of the body to cold depends on many factors including temperature, duration, area of exposure, and individual factors of the exposed person^16^. Furthermore, BAT metabolism is affected by various factors: physiological (e.g. age, gender, body fat level), environmental (e.g. temperature, hypoxia), and diseases factors (e.g. diabetes mellitus)^15^. Among the factors regulating BAT functions, endogenous hormones (e.g. myokines: irisin and myostatin [Mst]) and substances known as BATokines (e.g. interleukin-6 [IL-6]) have been reported^15^.

Irisin is a thermogenic protein responsible for the conversion of white adipose tissue to BAT^17^. Several factors alter the level of circulating irisin including physical activity and cold temperatures. Irisin influences skeletal muscle, bone, the pancreas and liver, and it enhances insulin sensitivity, metabolism, and osteogenesis^18^. Mst is a member of the transforming growth factor beta (TGF-β) superfamily that regulates skeletal muscle mass^19^. Mst-deficient mice show an increase in skeletal muscle mass^20^. Mst, beyond its effect on skeletal muscle, may be involved in the control of energy balance^21^. IL-6 is a multifunctional protein produced by immune cells of the endothelium, connective tissue, adipose tissue, and skeletal muscle^22^. Factors inducing the expression of the IL-6 gene in skeletal muscle include reactive oxygen species^23^.

Our previous studies showed that exposure to hypobaric hypoxia for 2 weeks during a climbing expedition decreased the level of 25-hydroxyvitamin D (25(OH)D) in the human body^24,25^. Other authors have not unequivocally concluded whether the reason for this decrease in 25(OH)D levels in blood serum is due to hypoxia and the related production of pro-inflammatory factors, exercise-induced muscle damage, or/and low ambient temperatures. Moreover, athletes are at risk of vitamin D deficiencies; therefore, according to the recommendations of the Australian Institute of Sport^26^, vitamin D supplementation may be necessary to ensure optimal bone health, mitigate injury risk, and improve sports performance.

It should be noted that despite the growing number of studies on the effects of WBC on the body, the possible impact of cryotherapy on vitamin D levels has not been determined despite the anti-inflammatory, immunomodulatory, antioxidant, and anti-fibrotic properties of vitamin D^27^. In addition to vitamin D, adipose tissue and skeletal muscle, which secrete molecules such as cytokines, myokines, and BATokines, play an important role in maintaining the metabolic homeostasis of the body. In view of their involvement in the thermogenesis process, we aimed to investigate the effects of repeated exposure to systemic cryotherapy at −110 °C in physically active volunteers with different physical fitness levels on 25(OH)D, myokines (irisin, Mst, and IL-6), myoglobin, and high-sensitivity C-reactive protein (hsCRP) levels. The response to cold was evaluated during the first and tenth procedures. Solianik *et al*.^16^ demonstrated gender-specific neuroendocrine and immune responses to cold; therefore, only lean young men were enrolled in the study.

## Results

### Pre-treatment data

Pre-therapy data are presented in Table 1. Participants‘ demographics, excluding maximal oxygen consumption (VO_2_ max) levels, were comparable between the 2 groups.

**Table 1:** Pre-therapy somatic and physiological parameters of young healthy men.

	HPhL group	LPhL group	*p-value*
Body height [cm]	180.1 ± 4.68	182.3 ± 5.09	0.298
Body mass [kg]	76.2 ± 5.47	74.6 ± 6.64	0.434
BMI [kg/m^2^]	23.5 ± 1.59	22.3 ± 2.33	0.187
Free fat mass [kg]	61.5 ± 4.10	59.6 ± 4.42	0.294
Lean [kg]	58.3 ± 4.07	56.5 ± 4.15	0.334
Fat [%]	19.9 ± 4.49	20.1 ± 6.35	0.844
Fat mass [kg]	14.6 ± 3.86	14.5 ± 5.85	0.694
VO_2_ max [ml · kg^−1^ ·min^−1^]	49.9 ± 4.74	38.6 ± 3.01	<0.001

Data are presented as mean ± SD.

### Effect of 10 WBC sessions

Table 2 presents the values of biochemical indicators in both groups before the first and after the tenth cryotherapy session (30 minutes and following a 24-hour recovery).

**Table 2:** Effects of 10 sessions of WBC on biochemical indices in both investigated groups.

	HPhL group	LPhL group	*p*-value between groups
**Myoglobin [ng/mL]**			
before first	251.8 ± 154.48	236.5 ± 132.00	0.847
30 minutes after tenth	253.3 ± 123.57	202.7 ± 166.69	0.217
*p*-value (1 vs 5)	ns	ns	
24 hours after tenth	237.8 ± 134.27	183.56 ± 119.80	0.470
*p*-value (5 vs 6)	ns	ns	
*p*-value (1 vs 6)	ns	ns	
**hsCRP [U/L]**			
before first	0.57 ± 0.719	0.37 ± 0.274	0.646
30 minutes after tenth	1.38 ± 1.660	0.79 ± 0.747	0.300
*p*-value (1 vs 5)	ns	ns	
24 hours after tenth	1.20 ± 1.728	0.66 ± 0.577	0.646
*p*-value (5 vs 6)	ns	ns	
*p*-value (1 vs 6)	ns	ns	
**hsIL-6 [pg/mL]**			
Before first	0.96 ± 0.355	1.29 ± 1.133	0.793
30 minutes after tenth	1.12 ± 0.622	1.19 ± 0.493	0.511
*p*-value (1 vs 5)	ns	ns	
24 hours after tenth	1.14 ± 0.925	1.31 ± 0.624	0.212
*p*-value (5 vs 6)	ns	ns	
*p*-value (1 vs 6)	ns	ns	
**Irisin [ng/mL]**			
Before first	13.1 ± 3.28	14.1 ± 3.13	0.078
30 minutes after tenth	14.1 ± 5.57	15.3 ± 4.48	0.579
*p*-value (1 vs 5)	ns	ns	
24 hours after tenth	15.3 ± 3.39	16.3 ± 4.64	0.554
*p*-value (5 vs 6)	ns	ns	
*p*-value (1 vs 6)	ns	ns	
**Myostatin [ng/mL]**			
Before first	34.8 ± 5.49	29.3 ± 4.26	0.063
30 minutes after tenth	34.9 ± 5.62	28.7 ± 2.84	0.004
p-value (1 vs 5)	ns	ns	
24 hours after tenth	36.2 ± 5.92	31.3 ± 3.72	0.030
*p*-value (5 vs 6)	ns	0.043	
*p*-value (1 vs 6)	ns	ns	
**25(OH)D [ng/mL]**			
before first	28.3 ± 5.20	33.3 ± 5.42	0.339
30 minutes after tenth	31.3 ± 4.88	32.7 ± 4.98	0.497
*p*-value (1 vs 5)	0.002	0.020	
24 hours after tenth	30.1 ± 5.26	31.5 ± 5.48	0.559
*p*-value (5 vs 6)	ns	ns	
*p*-value (1 vs 6)	ns	ns	

Data are presented as mean ± SD. 1 = before first WBC, 5 = 30 minutes after tenth WBC, and 6 = 24 h after tenth WBC.

Immediately after 10 days of cryotherapy, significant changes in 25(OH)D levels in both groups were found compared to baseline levels: an increase in the high physical fitness level (HPhL) group (p = 0.002) and a decrease in the low physical fitness level (LPhL) group (p = 0.020). After 24 hours, a non-significant reduction in 25(OH)D levels in both groups were noted, as compared to baseline levels.

No significant differences in biochemical variables were found between the groups, except for Mst after 10 days of cryotherapy (30 minutes after p = 0.004; 24 hours after the last session p = 0.030). Significant changes in Mst concentration were observed in response to 10 WBC sessions in the LPhL group (p _*ANOVA*_ = 0.041), and there was a significant increase in the level of Mst 24 hours after the final WBC session compared to the value noted 30 minutes after completion (p = 0.043).

Table 3 presents the results of the correlation analysis, which was performed in each group by determining the correlation in the changes of biochemical parameters before the first WBC session and 30 minutes after (Δ_1–5_) and 24 hours (Δ_1–6_) after the last treatment. In the LPhL group, positive correlations were found between changes (Δ_1–5_) in the levels of 25(OH)D and Mst (r = 0.61; p = 0.46) and hsIL-6 and hsCRP (r = 0.69; p = 0.019). In the HPhL group, changes (Δ_1–6_) in concentrations of irisin were negative correlated with changes in hsIL-6 levels (r = −0.74; p = 0.010).

**Table 3:** Spearman’s rank correlation coefficients of tested variables.

Variables	HPhL group		LPhL group	
	r	*p-*value	r	*p-*value
Δ_1–2_ 25(OH)D/Δ_1–2_ hsCRP	−0.60	0.049		
Δ_1–2_ Irisin/Δ_1–2_ hsIL-6	−0.64	0.035		
Δ_1–3_ hsCRP/Δ_1–3_ Myoglobin	0.77	0.005		
Δ_4–5_ 25(OH)D/Δ_4–5_ Irisin	−0.70	0.017		
Δ_4–5_ 25(OH)D/Δ_4–5_ Myostatin			0.63	0.037
Δ_4–5_ IL-6/Δ_4–5_ hsCRP			0.69	0.019
Δ_1–5_ 25(OH)D/Δ_1–5_ Myostatin			0.61	0.046
Δ_1–5_ IL-6/Δ_1–5_ Myoglobin			0.66	0.026
Δ_1–6_ Irisin/Δ_1–6_ hsIL-6	−0.74	0.010		

1 = before first WBC, 2 = 30 minutes after first WBC, 3 = 24 h after first WBC, 4 = before tenth WBC, 5 = 30 minutes after tenth WBC, and 6 = 24 h after tenth WBC.

### Effect of single stimulus (first and tenth WBC sessions)

**Figure 1:**
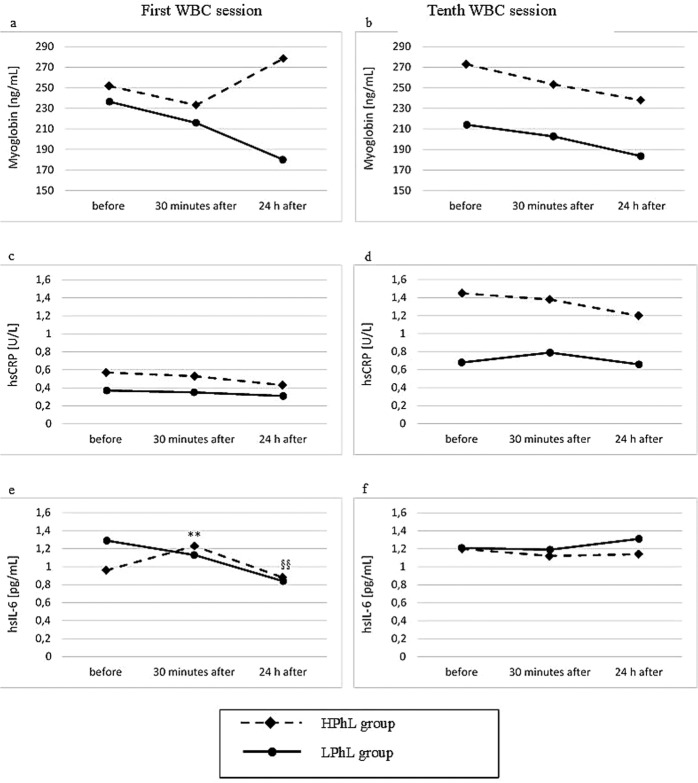
Blood concentrations of myoglobin, hsCRP, and hsIL-6 in young healthy men (first and tenth cryostimulations) (**a**,**b**) myoglobin (**c**,**d**) hsCRP (**e**,**f**) hsIL-6 **p < 0.01 significant differences between measurements before and 30 minutes after the first cryostimulation ^§§^p < 0.01 significant differences between measurements 30 minutes and 24 hours after the first cryostimulation.

**Figure 2:**
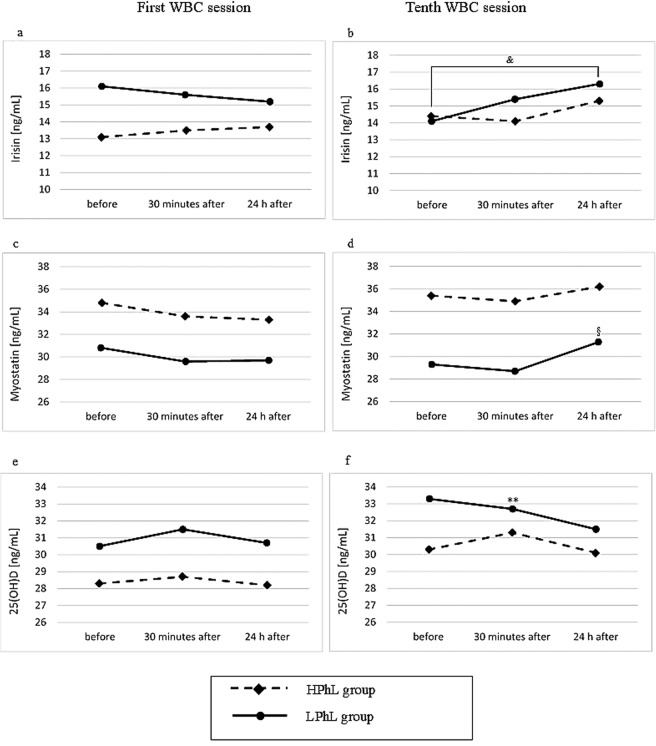
Blood concentrations of irisin, myostatin, and 25(OH)D in young healthy men (first and tenth cryostimulations) (**a**,**b**) irisin (**c**,**d**) myostatin (**e**,**f**) 25(OH)D **p ≤ 0.01 significant differences between measurements before and 30 minutes after the first cryostimulation ^&^p < 0.05 significant differences between measurements before and 24 hours after the first cryostimulation ^§^p < 0.05 significant differences between measurements 30 minutes and 24 hours after the first cryostimulation.

Figures 1 and 2 present the values of biochemical indicators in both groups before, 30 minutes and 24 hours after the first, and after the tenth cryotherapy sessions.

The HPhL group showed significant changes in the level of hsIL-6 after the first treatment (p _*ANOVA*_ = 0.001); 30 minutes after cryotherapy, there was a significant increase compared to baseline values (p = 0.007), and after 24 hours, a decrease was observed compared to the value observed 30 minutes after therapy (p = 0.001).

Moreover, in the LPhL group, significant changes in the levels of 25(OH)D, irisin, and Mst were noted after the tenth treatment (p_*ANOVA*_ = 0.025, p_*ANOVA*_ = 0.045, and p_*ANOVA*_ = 0.041, respectively). The level of 25(OH)D significantly decreased 30 minutes after cryotherapy compared to baseline (p = 0.010). Additionally, the level of irisin significantly increased 24 hours after cryotherapy compared to baseline values (p = 0.036), whereas the Mst concentration increased 24 h after cryotherapy compared to 30 minutes after (p = 0.043).

Table 3 presents the results of the correlation analysis for each group after the first and the tenth cryotherapy sessions in the changes observed in biochemical indices before and 30 minutes after the treatment, as well as 24 hours after cryostimulation. After the first cryotherapy session, negative correlations were found between changes (Δ_1–2_) in the levels of 25(OH)D and hsCRP (r = −0.60; p = 0.049), as well as irisin and hsIL-6 (r = −0.64; p = 0.035) only in the HPhL group. A positive correlation was observed between changes (Δ_1–3_) in hsCRP and myoglobin concentrations.

After the tenth cryotherapy session, changes (Δ_4-5_) in 25(OH)D levels were negative correlated with changes (Δ_4-5_) in irisin levels (r = −0.70; p = 0.017) in the HPhL group, whereas in the LPhL group, positive relationships were found between changes (Δ_4-5_) in the levels of 25(OH)D and Mst (r = 0.63; p = 0.037), as well as hsIL-6 and hsCRP (r = 0.64; p = 0.035).

## Discussion

To the best of our knowledge, this is the first study assessing the effect of WBC on vitamin D status. We found significant changes in 25(OH)D levels in both groups 30 minutes after 10 days of cryotherapy, compared to pre-therapy levels (Table 2). We found an increase in the HPhL group and a decrease in the LPhL group. We also observed a further slight decrease in the serum 25(OH)D level 24 hours after the tenth WBC stimulus compared to the pre-therapy values, exclusively in the LPhL group (Table 2). It should be noted that one might expect a small increase in 25(OH)D concentration during the few days in the middle of June in which the research was conducted, because, as observed Osmancevic *et al*.^28^, it takes approximately 7 days for serum 25(OH)D levels to peak after ultraviolet B exposure. As Poland has a latitude of 49–54°N, the solar angle and weather conditions suitable for vitamin D synthesis occur between late April and early September^29^. Some studies have indicated that the exposed skin area and the baseline vitamin D status also affect the synthesis of cholecalciferol in the skin. Osmancevic *et al*.^28^ found that exposure of smaller body surface areas, such as the face and hands (while study participants are wearing a T-shirt and shorts), induces less vitamin D production. Moreover, other studies showed that current sun-exposure practices of the general population do not provide sufficient amounts of vitamin D^30,31^.

Despite not finding any studies on the effect of cryotherapy on the level of vitamin D in the available literature, we presume that the observed changes in 25(OH)D levels might be attributed to the role of vitamin D in the inflammatory response, which is indicated by the inverse relationship between changes (Δ_1–2_) in concentrations of 25(OH)D and hsCRP, noted 30 minutes after the first WBC treatment in the HPhL group (Table 3). Studies have shown that vitamin D may possess natural antioxidant and anti-inflammatory properties^24,32,33^.

Moreover, our study shows that a series of 10 WBC sessions contributed to diverse systemic thermogenic responses depending on the level of physical fitness of the study participants.

**Figure 3:**
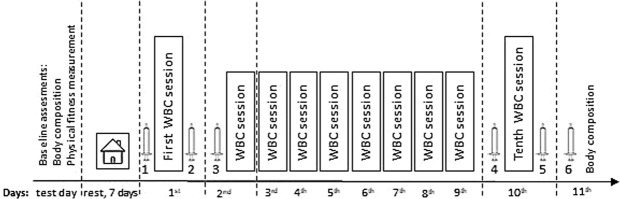
Experiment schedule WBC = whole-body cryotherapy  = blood collection = before first WBC, 2 = 30 minutes after first WBC, 3 = 24 h after first WBC, 4 = before tenth WBC, 5 = 30 minutes after tenth WBC, and 6 = 24 h after tenth WBC.

We found an increase in serum irisin after 10 days of therapy compared to the pre-therapy level (Table 2), which was not statistically significant (17% in the HPhL group and 16% in the LPhL group). In the LPhL group, there was a significant increase 24 hours after the tenth application compared to the level observed before the application of WBC (Fig. 3).

Our results are partly comparable to those reported by Dulian *et al*.^34^, who found a 20% increase in the plasma irisin concentrations in middle-aged, obese, non-active men and a slight decrease in active subjects 24 hours after 10 WBC sessions. The authors suggested that an increase in the level of irisin following exposure to a low ambient temperature may be induced by shivering thermogenesis, which causes irisin secretion from skeletal muscle. However, the inverse relationship between the muscle mass and irisin concentration obtained by Dulian *et al*.^34^ may point to the subcutaneous adipose tissue as the source of irisin.

Moreno-Navarrete *et al*.^35^ and Roca-Rivada *et al*.^36^ also showed that irisin was secreted not only from the muscles, but also from the visceral and subcutaneous adipose tissue. No such correlations were found in our study; however, it must be noted that only lean men were enrolled in our study, whereas the authors cited above conducted their studies on individuals with an elevated body fat percentage.

In our study, we evaluated the effects of WBC treatments on the level of Mst, which plays an important role in regulating energy homeostasis through the modulation of skeletal muscle mass^37^. Previous research findings suggested that inhibition of the TGF-β/Mst superfamily increases the activity of BAT, which leads to increased energy expenditure and provides metabolic benefits^21^.

Our results showed significant changes in Mst concentration only in the LPhL group 24 hours after the tenth WBC session compared to the values found 30 minutes after completing these session (Fig. 3). However, Mst concentration did not differ significantly from pre-therapy values. Our results are consistent with that of the available literature stating that changes in the Mst levels induced by environmental stimuli and/or physical exercise are transient and return to the baseline level within 24 hours after the application of the triggering factor^38^. In turn, the activation of satellite cells inducing inflammation requires more than 24 hours^39^.

In the LPhL group, we found positive correlations between changes in levels of 25(OH)D and Mst 30 minutes after the tenth WBC treatment (Δ_4–5_) and compared to baseline (Δ_1–5_) (Table 3). These results, as well as the negative correlations between changes (Δ_4–5_) in concentrations of 25(OH)D and irisin in the HPhL group (Table 3), confirmed the role of vitamin D in skeletal muscle metabolism. Slivka *et al*.^40^ found that the induction of peroxisome proliferator-activated receptor (PPAR)-γ Coactivator (PGC-1α) gene expression is enhanced after exercise in a cold environment. Irisin is secreted in response to PGC-1α^17^, which may act as a co-activator of the vitamin D receptor in mitochondria^41^. Furthermore, Garcia *et al*.^42^ showed that vitamin D suppresses the expression of Mst, while it up-regulates the expression of follistatin and insulin-like growth factor II.

A considerable number of studies point to the anti-inflammatory effect of WBC^1,3,43,44^; therefore, we evaluated the levels of hsCRP and IL-6. In recent years, studies have indicated that Mst stimulates IL-6 production in muscle cells and adipose tissue^45^; however, in our study, we did not find a significant relationship between these molecules. It should be noted that in our study, blood was collected after physical exercise, which was performed immediately after WBC treatment. As shown by Rhind *et al*.^46^, there is a lower expression of genes encoding for proinflammatory cytokines in young healthy volunteers immediately after the training preceded by exposure to low temperatures, compared to training alone.

In the HPhL group, a significant change in the concentration of IL-6 was noted only after the first WBC stimulus (Fig. 2). No statistically significant changes after 10 days of cryotherapy were found. Our findings are consistent with the results previously published by Lubkowska *et al*.^47^, who showed that a series of WBC sessions did not trigger an increase in the concentration of proinflammatory cytokines in healthy young men. On the other hand, Ziemann *et al*.^48^ found a decrease in tumour necrosis factor alpha with a concurrent increase in IL-6 after 5 days of treatments conducted twice a day in conjunction with moderate-intensity training in professional athletes. This finding was explained by the fact that WBC significantly lowered the inflammatory response induced by eccentric exercise. The inverse relationship between changes (Δ_1–2_ and Δ_1–6_) in the concentration of irisin and IL-6, noted by us in the HPhL group (Table 3), points to the anti-inflammatory characteristics of irisin, which is consistent with the findings of Mazur-Biały *et al*.^49^.

Our study has some potential limitations. In particular, a small number of subjects participated in this study. Additionally, the study period was too short to observe the long-term effects of WBC. There was also a lack of standardised treatment protocols with regard to temperature ranges, timing, and frequency of exposure to WBC, which is likely to elicit varying recovery responses to the therapy.

Our data demonstrated that in healthy young men with a normal body weight, cryotherapy affects levels of 25(OH)D. The body’s response to a series of 10 cryotherapy treatments is modified by the physical fitness level. It should be noted that the observed changes in the serum 25(OH)D were small and transient, therefore athletes can use WBC treatments. However, in order to fully explain the body’s response to cryostimulation, additional studies need to be performed on a larger study group.

## Methods

### Study group

This study was conducted in June over 10 consecutive days. The study design and timeline are presented in Fig. 3. Twenty-six volunteers (students at the Universities) were initially enrolled in this study. The inclusion criteria for the study were as follows: healthy males, age 19–23 years, body mass index (BMI) > 18.5 and <25.0, non-smokers, had not previously undergone systemic cryotherapy, and subjects who provided written consent. The exclusion criteria were as follows: contraindication to WBC and supplementation with vitamin D.

In total, 22 subjects (mean age: 21 ± 1.17 years) completed the entire study protocol and were included in the analysis. Some studies suggested that physical fitness level might modify the effect of cryotherapy^34,50^. Therefore, the participants performed a treadmill exercise test, and based on the classification of VO_2_ max proposed by Astrand^51^, they were divided into 2 groups: group 1, HPhL with a higher VO_2_ max ≥43 (n = 11); and group 2, LPhL with a lower VO_2_ max < 43 (n = 11).

For 48 hours before the tests, the participants did not perform intense physical exercises that could lead to dehydration and delayed onset muscle soreness (DOMS). During the experiment, all participants were instructed not to change any aspect of their habits, such as diet, and to avoid any form of exercise.

Each participant was familiarised with all testing procedures and provided written informed consent prior to the study. The study protocol was approved by the Ethics Committee for Human Research at the Poznań University of Medical Sciences (approval no. 572/2014) and was performed in accordance with the Declaration of Helsinki. Thus, all participants were informed about the methodology of the experiment methodology, the risks of the treatment, and the opportunity to withdraw from the study at any point without providing any reason.

### Body composition assessment

One week prior to the start of the experiment, the body composition and aerobic capacity of each participant were measured. Body mass and body height were measured using a certified medical digital beam scale WB-3000 (TANITA Corporation, Tokyo, Japan), with an accuracy of 0.01 kg, and a mechanical measuring rod for body height HR-001 (TANITA Corporation, Tokyo, Japan), with an accuracy of 0.5 cm. Body composition was measured in the fasting state using a GE Lunar Prodigy Primo Full Densitometer with enCore Body Composition option (GE Healthcare Technologies, USA). BMI was calculated by dividing body mass (kg) by the square of body height (m^2^).

### Physical fitness measurement

One week before the start of the experiment, the participants performed a treadmill exercise test (HP Cosmos Saturn, Germany). The test was initiated at a baseline speed of 6 km/h and then continuously increased. At 3-minute intervals, the speed of the treadmill was increased by 2 km/h, up to the maximal speed for a given subject characterised by the lack of increase in minute oxygen uptake despite increased exercise intensity. The test was not preceded by a warm-up session. Circulation and respiratory parameters were monitored continuously using an ergospirometer (VO_2 max_ Finder, MES, Poland). Heart rate was registered every 5 seconds with a Polar Accurex Plus device (Polar Elektro, Finland).

### Whole-body cryostimulation

All participants underwent a series of exposures to cold (once a day between 8:00–10:00 a.m.) for a total of 10 sessions in a cryogenic chamber (Zimmer Ice Lab, −110 °C, Medizin System GmbH; Germany). Each exposure was preceded by a light breakfast between 7:00–7:30 a.m. according to the instructions given to the subjects.

Each cryostimulation session lasted 3 minutes at −110 °C. The entry into the cryochamber was preceded by a 20–30-second period of adaptation in the vestibule at −60 °C. The subjects were dressed in shorts, socks, gloves, and a headband covering their ears. Following WBC, the participants performed physical exercise at 100 W on a cycloergometer (Keiser M3, Germany) for 15 minutes.

### Biochemical analyses

Blood samples for biochemical analyses were taken 6 times from the antecubital vein and centrifuged at 4000 rpm and 4 °C: before, 30 minutes after, and 24-hours after the first exposure; and before the final cryotherapy session and 30 minutes and 24-hours after recovery following the final cryotherapy session. The serum was separated from the sample and stored at −70 °C.

HsCRP and myoglobin levels were measured by immunoenzymatic assay using commercially available kits (DRG International Inc., Springfield Township, NJ, USA; test sensitivity: 0.1 mg/L and 5 ng/mL), as were the levels of hsIL6 (R&D Systems Inc., Minneapolis, MN, USA; test sensitivity: 0.039 pg/mL), irisin (Aviscera Bioscience Inc., Santa Clara, CA, USA; test sensitivity: 100 pg/mL), and Mst (Immundiagostik AG, Bensheim, Germany; test sensitivity: 0.37 ng/mL). The serum concentration of 25(OH)D was measured by chemiluminescent immunoassay (CLIA, DiaSorin Liaison, Stillwater, USA; test sensitivity: 4 ng/mL).

### Statistical analysis

Data were presented as means and standard deviations (SD). The Shapiro-Wilk test was used to check the data for normality of distribution. Assumption on sphericity was tested using Mauchley’s test, verifying if variances of certain variables were identical and equal to respective co-variances. The 1-way analysis of variance (ANOVA) with repeated measures was used to compare 1 quantitative variable with normal distribution at 3 points in time. When ANOVA showed significance, the post hoc Tukey’s honestly significant different test was applied to indicate which measurements tested at 3 time points of the study were significantly different. For data not normally distributed, the Friedman nonparametric test was used for comparison of repeated measured values over the study period at the 3 time points, followed by the Dunn’s post hoc test to detect differences between each time point. The statistical significance threshold was set at p < 0.05. Relationships between variables were tested using Spearman’s rank correlation. All analyses were performed using the Statistica 13.0 software package (StatSoft, Tulsa, Oklahoma, USA).

## Supplementary information

Supplementary Information.

## Data Availability

The datasets generated during and/or analysed during the current study are available from the corresponding author on reasonable request.
